# Left ventricular noncompaction in Ibadan, Nigeria

**DOI:** 10.1186/s43044-023-00396-9

**Published:** 2023-08-10

**Authors:** Okechukwu Samuel Ogah, Efosa P. Iyawe, Olanike Allison Orimolade, Kenechukwu Okwunze, Mesoma Okeke, Abdulhammed Babatunde, Akinyemi Aje, Adewole A. Adebiyi

**Affiliations:** 1 Cardiology Unit, Department of Medicine, Faculty of Clinical Sciences, College of Medicine,, University of Ibadan, Ibadan, Nigeria; 2 Cardiology Unit, Department of Medicine, University College Hospital, Ibadan, PMB 5116, Ibadan, Nigeria; 3 Alexander Brown Hall, College of Medicine, University of Ibadan, Ibadan, Nigeria

**Keywords:** Cardiomyopathy, Echocardiography, Heart failure, Noncompaction, Trabeculation

## Abstract

**Background:**

There has been an increase in the reporting of cases of left ventricular noncompaction (LVNC) cardiomyopathy in medical literature due to advances in medical imaging. Patients with LVNC may be asymptomatic or may present with arrhythmias, heart failure, thromboembolism or sudden death. LVNC is typically diagnosed by echocardiography, although there are higher-resolution cardiac imaging techniques such as cardiac magnetic resonance imaging (MRI) to make the diagnosis. The objective of the study is to report on a series of 9 cases of LVNC cardiomyopathy seen at the University College Hospital, Ibadan. Cases of LVNC seen between September 1, 2015 and July 31, 2022 in our echocardiography service  is being reported.

**Results:**

There were a total of 6 men and 3 women. Mean age at presentation was 52.89 ± 15.02 years. The most common mode of presentation was heart failure (6 patients). Hypertension was the most common comorbidity (6 patients). Three patients had an ejection fraction of less than 40% and the mean ratio of noncompacted to compacted segment at end-systole was 2.80 ± 0.48. The most common areas of trabecular localization were the LV lateral wall and the apex. Beta blockers were highly useful in the management of the patients.

**Conclusions:**

LVNC cardiomyopathy is not uncommon in our environment and a high index of suspicion is often required.

## Background

Left ventricular noncompaction (LVNC) cardiomyopathy belongs to the group of unclassified cardiomyopathies according to the European Society of Cardiology Working Group on Myocardial and Pericardial Diseases [1]. It is caused by the arrest of the compaction of the left ventricle during embryogenesis [2]. Clinically, it is characterized by the presence of numerous and prominent trabeculae with deep inter-trabecular recesses which communicate with the cavity of the left ventricle but not with the coronary arteries [3]. LVNC cardiomyopathy can occur in isolation or with other cardiac or non-cardiac conditions such as atrioventricular canal defects, bicuspid aortic valve and complex cyanotic congenital heart abnormalities [4]

There are limited reports of this condition in Africa. The first case in sub-Saharan Africa was reported in 2006 by Ker and Van De Maewe [5]. Reports from other countries then followed including a prospective study of 54 cases by Peters et al. in South Africa [6]. Reports of isolated LVNC have been made in patients of Nigerian origin [7], including in the paediatric age group [8]. In 2016, we reported a case of LVNC in a Nigerian male teenager who presented to the emergency department of the University College Hospital, Ibadan [9].

The aim of the present study was to report a predetermined series of LVNC seen at our cardiology unit between September 2015 and July 2022 and to estimate the prevalence.

## Methods

This is the report of a case series collected between September 1, 2015 and July 31, 2022. The study was conducted at the Cardiology Unit of the Department of Medicine, University College Hospital, Ibadan. The study adheres to all widely accepted ethical principles guiding human research. Ethical approval was obtained as part of the Ibadan Heart Failure Registry, and all patients gave informed verbal consent before recruitment. The diagnosis of LVNC was made in the presence of the following on echocardiography [10].

1. Multiple (> 3) left ventricular trabeculae;
2. Deep inter-trabecular recesses;
3. Doppler colour flow within the recesses and communicating with the left ventricular cavity and
4. Double-layered endocardial structure with the noncompacted to compacted segment ratio of more than 2 in end-systole.

A proforma was used for the collection of their socio-demographic information, clinical details, 12-lead ECG findings and echocardiography data. All echocardiography screening was performed according to the guidelines of the American Society of Echocardiography [11].

We obtained the left ventricular (LV) septal and posterior wall thickness as well as the LV internal dimensions in diastole and systole. LV ejection fraction was obtained using the modified Simpsons biplane method [12]. The right ventricular (RV) systolic function was evaluated using the tricuspid annular plane systolic excursion (TAPSE). The LV filling pattern was studied by Doppler interrogation of the mitral valve flow pattern. The RV systolic pressure was assessed using the tricuspid regurgitation flow spectrum. Finally, the 17-segment model of the LV was used for the localization of the noncompacted segment. Simple descriptive statistics was used for the analysis of the findings.

## Results

**Fig. 1:**
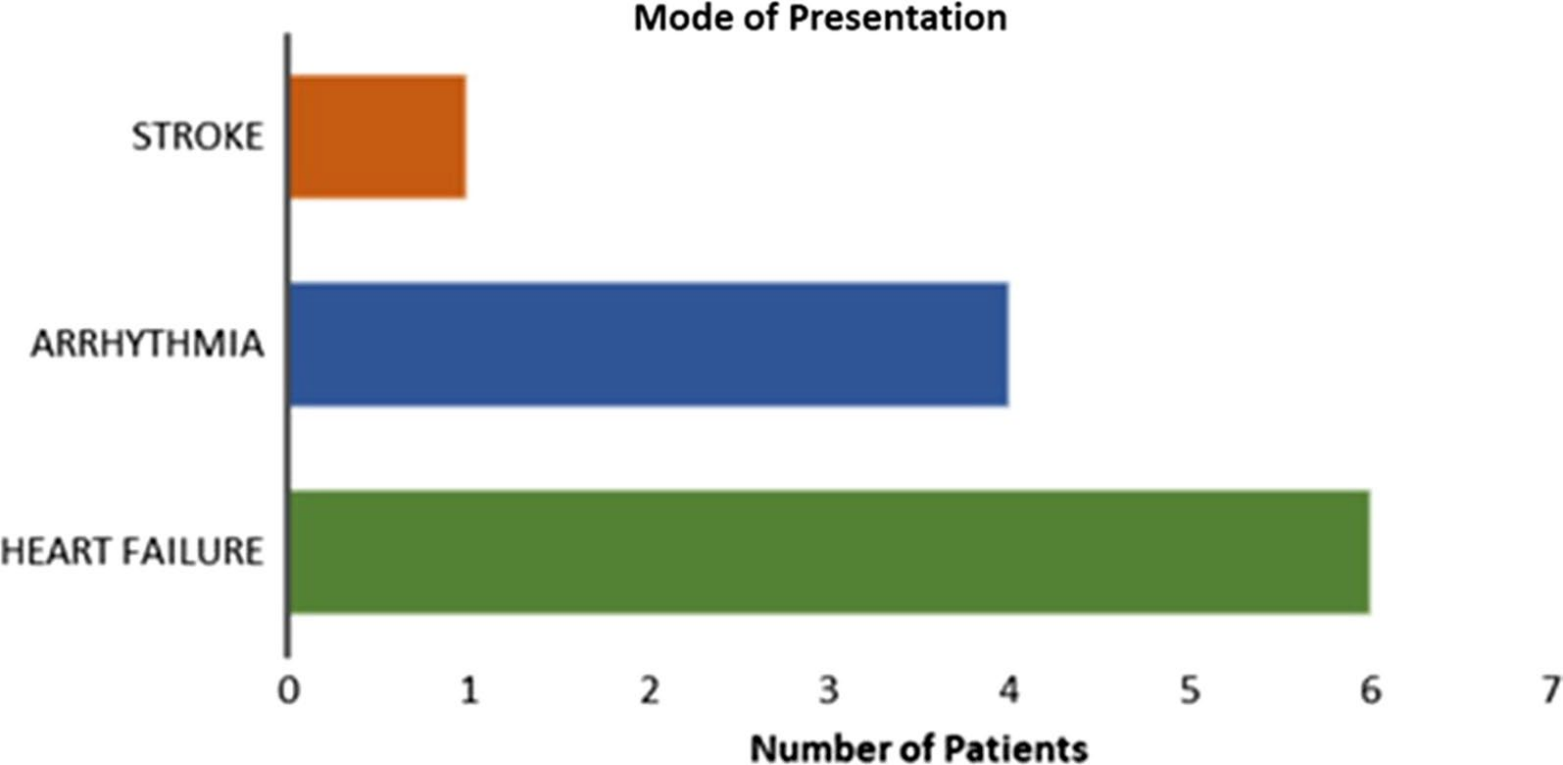
Shows the mode of presentation of the LVNC cases

During the study period, 14,949 patients had echocardiography procedures carried out in our laboratory and a total of nine patients met the case definition. Of these, 6 of them were male while 3 of them were females. Their ages ranged from 25 to 73 years, with the mean age being 52.89 ± 15.02 years. The clinical and electrocardiographic details of the patients are shown in Table 1. The modes of presentation observed in the nine patients include heart failure (6 patients), arrhythmia (4 patients) and ischaemic stroke (1 patient) (Fig. 1). Hypertension was the most common comorbidity observed in the patients (6 patients). The most common electrocardiographic findings were left axis deviation and left ventricular hypertrophy, each observed in 3 patients.

**Table 1:** Clinical and echocardiographic details of the cases

Case ID	Sex	Age	Presentation	Comorbidities	ECG findings	Medications
Case 1	Female	63	Arrhythmia	Hypokalaemia Hypertension Dyslipidaemia	LAHB 1st degree AV block RBBB	Amlodipine Amiodarone
Case 2	Male	30	Stroke	Intracardiac clot	LVH	Rivaroxaban
Case 3	Female	30	Heart Failure		LAE LVH with strain	Lisinopril Carvedilol Spironolactone Torsemide Digoxin Dapagliflozin
Case 4	Male	53	Heart Failure	Hypertension	LAD T-wave inversion in lateral leads	Telmisartan Rosuvastatin Spironolactone Beta blocker Furosemide Aspirin
Case 5	Male	67	Arrhythmia	Hypertension	1st degree AV block	Cardioversion Amlodipine Amiodarone
Case 6	Male	48	Heart Failure	Hypertension	LAE LVH with strain	Candesartan Clopidogrel Bisoprolol Rosuvastatin HCT
Case 7	Female	54	Heart Failure	Hypertension	LAD	ACE Inhibitor Beta blocker
Case 8	Male	73	Heart Failure Arrhythmia		Tachycardia Atrial Fibrillation LAD Poor R wave progression	Torsemide, Spironolactone Dapagliflozin
Case 9	Male	58	Heart Failure Arrhythmia	Hypertension Hypokalaemia	Q wave in V1-V3	Ramipril, Bisoprolol

*AV* Atrioventricular, *ECG* Electrocardiography, *LAD* Left Axis Deviation, *LAE* Left Atrial Enlargement, *LAHB* Left Anterior Hemiblock, *LVH* Left Ventricular Hypertrophy, *RBBB* Right Bundle Branch Block

On echocardiography, the mean ejection fraction (EF) was 49.18 ± 17.49%, with 3 of the patients having an EF less than 40%. Up to six patients had reduced fractional shortening (< 29%). The mean ratio of noncompacted to compacted segments (NC/C) at end-systole was 2.80 ± 0.48 (Table 2). The most common areas of localization were apical and left ventricular wall (7 patients each), while only one patient had right ventricular involvement. Mitral regurgitation was found in 4 patients, 3 patients had tricuspid regurgitation, and 3 patients had wall motion abnormalities. The echocardiographic findings of the patients are summarized in Tables 3 and 4. A variety of medications were used across the nine patients. The most common medications used were beta blockers (5 patients), ACE inhibitors (3 patients), loop diuretics (3 patients) and spironolactone (3 patients).

**Table 2:** Trabecular localization of the cases

Case ID	LV lateral	LV posterior	Apex	Septum			RV wall	Comments
				Apical	Mid	Basal		
Case 1	Present	–	Present	Present	Present	Present	–	Frequent ectopics, Mild PE, Apical hypokinesis
Case 2	Present	–	Present	–	Present	–	–	–
Case 3	Present	–	Present	Present	**–**	**–**	Present	MR, TR, PR
Case 4	Present	–	Present	Present	Present	**–**	**–**	Global hypokinesis, MR, TR
Case 5	**–**	Present	Present	**–**	**–**	**–**	**–**	**–**
Case 6	Present	**–**	Present	**–**	Present	Present	**–**	MR
Case 7	Present	**–**	Present	**–**	Present	Present	**–**	**–**
Case 8	Present	**–**	**–**	Present	**–**	**–**	**–**	Global hypokinesis, MR, TR

*LV* Left ventricle, *MR* Mitral Regurgitation, *PR* Pulmonary Regurgitation, *RV* Right ventricle, *TR* Tricuspid Regurgitation

**Table 3:** Echocardiographic details of the cases

Case ID	LA	LVID	LVIS	IVSD	PWTD	TAPSE	E/A	RVSP	FS	EF	NC/C_d_	NC/C_S_
1	3.3	5.2	3.8	0.8	1.4	1.8	0.62	–	28	53	2.2	2.6
2	3.7	5.6	3.6	1.2	0.8	2.7	1.28	–	36.5	65.6	1.1	3.5
3	4.7	6.9	5.9	0.9	1.0	1.22	2.76	40.9	15	30	2.2	2.0
4	5.4	6.6	5.4	1.1	0.8	2.6	3.29	–	16	33	1.0	3.0
5	3.8	5.1	3.2	0.8	1.2	3.1	1.02	–	38	68	0.9	3.3
6	4.4	6.6	5.2	1.0	0.8	2.0	–	24.3	21	42	2.3	2.4
7	3.2	4.5	3.0	0.8	0.8	2.5	0.96	14.4	28	54	2.5	2.4
8	4.6	6.3	5.5	0.9	1.0	1.6	–	40.0	12	25	1.0	3.0
9	4.9	5.5	3.2	1.2	1.5	2.2	0.73	–	42	72	2.5	3.0

*E/A * Transmitral E to A wave ratio E/A, *EF* Ejection fraction (%), *FS* Fractional shortening (%), *IVSD* Interventricular septal wall thickness in diastole (cm), *LA* Left Atrial Diameter (cm), *LVID* Left ventricular internal dimension in diastole (cm), *LVIS* Left ventricular internal dimension in systole (cm), *NC/C*_*d*_ Ratio of noncompacted to compacted segment in diastole, *NC/C*_*S*_ Ratio of noncompacted to compacted segment in systole, *PWTD* Left ventricular posterior wall thickness in diastole (cm), *RVSP* Estimated right ventricular systolic pressure (mmHg), *TAPSE* Tricuspid annular plane systolic excursion (cm)

**Table 4:** Mean echocardiographic parameters of the cases

Echocardiographic parameters	Mean (*n* = 9)
Left atrial diameter (cm)	4.22 ± 0.76
Left ventricular internal dimension in diastole (cm)	5.81 ± 0.82
Left ventricular internal dimension in systole (cm)	4.30 ± 1.17
Interventricular septal wall thickness in diastole (cm)	0.97 ± 0.17
Left ventricular posterior wall thickness in diastole (cm)	1.03 ± 0.27
Tricuspid annular plane systolic excursion (cm)	2.19 ± 0.60
Transmitral E to A wave ratio E/A (*n* = 7)	1.52 ± 1.06
Estimated right ventricular systolic pressure (mmHg) (*n* = 4)	29.90 ± 12.84
Fractional shortening (%)	26.28 ± 10.95
Ejection fraction (%)	49.18 ± 17.49
Ratio of noncompacted to compacted segment in diastole	1.74 ± 0.72
Ratio of noncompacted to compacted segment in systole	2.80 ± 0.48

## Discussion

During embryogenesis, a normal compaction process occurs in the ventricular myocardium in order to convert it into a denser consistency. Failure of this process to occur leads to left ventricular noncompaction with numerous trabeculations and deep recesses between them [2] Various genetic defects have been associated with LVNC, including mutations in the Z-band alternatively spliced PDZ-motif protein (ZASP) gene on chromosome 10 [10, 13] These mutations could be sporadic or familial. Hence, screening is recommended for individuals with a family history [14].

Due to the advent of better imaging techniques, there has been an upsurge in the diagnosis of LVNC [15] Although the prevalence varies with imaging modality used [16], it has generally been found to be more prevalent in the African population [2, 17]

A number of studies have been reported in Africa [5, 9, 18], but there is still a paucity of literature on characteristics of patients with LVNC in the region. Our study was conducted in Nigeria, one of the countries in sub-Saharan Africa.

LVNC has been shown to have a male gender predilection [18], and this was evident in our study, with two-thirds of the participants being males. Previously, LVNC was thought to be a disease of the young, but it was later described across older age groups and has now been found to occur at any age [19]. This is similar to what was found in this study as the patients’ ages ranged from 25 to 73 years. The mode of presentation of LVNC varies [18, 20] Patients may be asymptomatic but most (67%) cases present with systolic heart failure while other modes of presentation include arrhythmia and thromboembolic events [18]. Similarly, the most common presentation in this study was heart failure, seen in 6 patients (66.7%), two of which had coexisting arrhythmia. Although systolic heart failure is common with LVNC [19], only 3 of the patients had an ejection fraction of less than 40%. Ali [18] and Oechslin et al. [21] reported arrhythmia in 14% and 41% and thromboembolism in 9% and 24%, respectively, in their series. The thromboembolic events are usually due to the trabeculations, systolic dysfunction and atrial fibrillation [22, 23]. In our study, 4 patients presented with arrhythmia, while only one patient had an ischaemic stroke.

The electrocardiographic findings in LVNC vary depending on the presence of arrhythmia and its type. Atrioventricular re-entrant tachycardia and ventricular tachycardia are more common in the young, while atrial fibrillation and ventricular arrhythmias are more common in older adults [22, 24]. Other electrocardiographic features associated with LVNC include bundle branch block, fascicular block, atrioventricular (AV) block and T-wave inversions [25, 26]. In our study, first-degree AV block was found in 2 patients, while right bundle branch block, left anterior hemiblock and T-wave inversion were found in one patient each.

The most sensitive modality for diagnosing LVNC is the use of cardiac magnetic resonance imaging (CMRI). It helps provide better visualization of the trabeculations, especially at the apical and anterolateral regions of the ventricle [27]. However, asides being more expensive, CMRI requires more expertise and patient stability. MRI requires that patients are able to lie down flat and hold their breath, which may be difficult for patients with heart failure. Therefore, two-dimensional echocardiography with colour Doppler is the first-line investigation for LVNC [28]. Furthermore, in developing countries like Nigeria where cost of investigation is an issue, trans-thoracic echocardiography is the most cost-effective method of investigation and was used in this study.

Several diagnostic criteria have been put forward for the diagnosis of LVNC [10, 21, 29]^.^ Most of them use echocardiography, with the most popular being the one by Jenni et al. [10] which was the criteria used in our study. One of the patients had an NC/C ratio of exactly 2.0 at end-systole, but met all the other criteria, hence was still included in the study. The other patients had their NC/C ratio in end-systole well over 2.0, with the average ratio being 2.8.

**Fig. 2:**
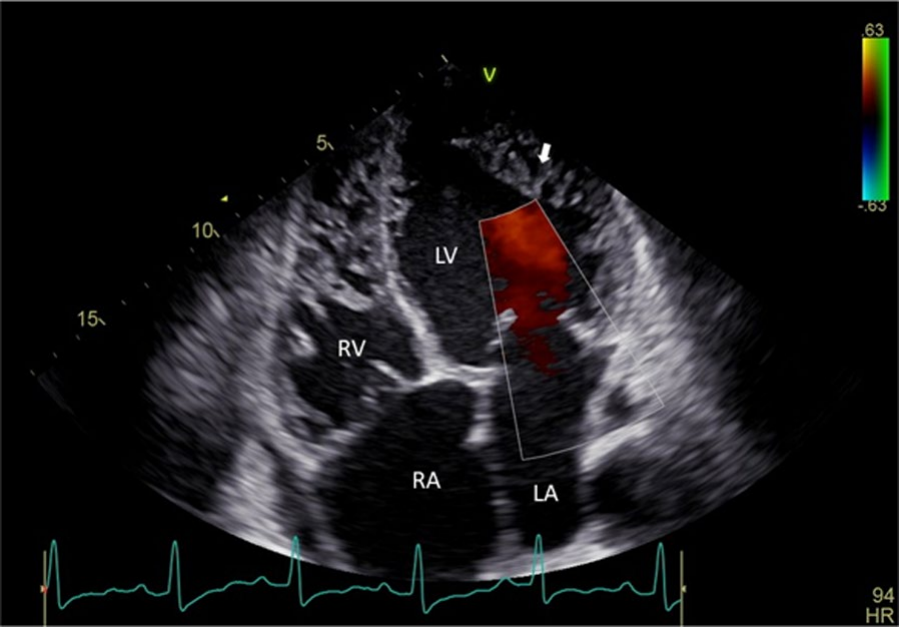
Shows trabeculations and colour flow in the recesses of left ventricle

The localization of the trabeculae is important in symptomatology. In patients with LVNC, the trabeculae are most commonly found in the apex and lateral walls [5, 9, 30]. Comparably, the most common areas of trabecular localization in this study were apical and left ventricular lateral wall (7 patients each) (Fig. 2). The least involved areas were left ventricular posterior wall and right ventricle (1 patient each). Biventricular involvement is rare in LVNC [31] as was seen in this study with only one patient having biventricular involvement. It has been observed that further review of the echocardiography of patients with LVNC may show left ventricular systolic dysfunction with reduced ejection fraction [30] as was seen in 3 of our patients. Other cardiac abnormalities which may be seen in patients with LVNC include regional wall motion abnormalities, especially in the areas of trabeculations [31]. This was seen in 3 of our patients, two of which had global hypokinesis, while one had apical hypokinesis.

Treatment of patients with LVNC varies depending on the mode of presentation, comorbidities and complications developed. Heart failure is managed with ACE inhibitors, beta blockers and diuretics, rhythm abnormalities are contained depending on the type of arrhythmia [32]. The use of anticoagulation for thromboembolism varies among authors. Some authors believe that anticoagulation be given to patients with LVNC whether or not they have a history of thrombosis [10, 22, 23]^.^ Beta blockers were used in 5 of the patients to control heart failure, while 3 patients each received ACE inhibitors, loop diuretics and spironolactone. Amiodarone was used for rate control in two patients, while one of them received electrical rate control by cardioversion.

The mortality rate of patients with LVNC is about 35%. The most common causes of death in these patients are sudden cardiac death causing up to half of deaths, while heart failure causes about one-third of deaths [22]. Sudden cardiac death could be due to heart failure, arrhythmias or thromboembolic phenomena [33, 34]. Follow-up of the study participants could reveal the mortality rate in this study, as well as the causes of mortality. This could be used as a proxy to the common causes of mortality among patients with LVNC in this environment.

This study is the first report of LVNC case series in Nigeria. This study highlights that the disease is not uncommon in our environment and may be underreported due to poor imaging techniques or poor index of suspicion. It is possible some cases were missed during the recruitment process due to the fact that cardiac MRI is not available in our centre. This imaging technique would have been very useful in ensuring a higher sensitivity and better characterization of the condition.

## Conclusions

**Fig. 3:**
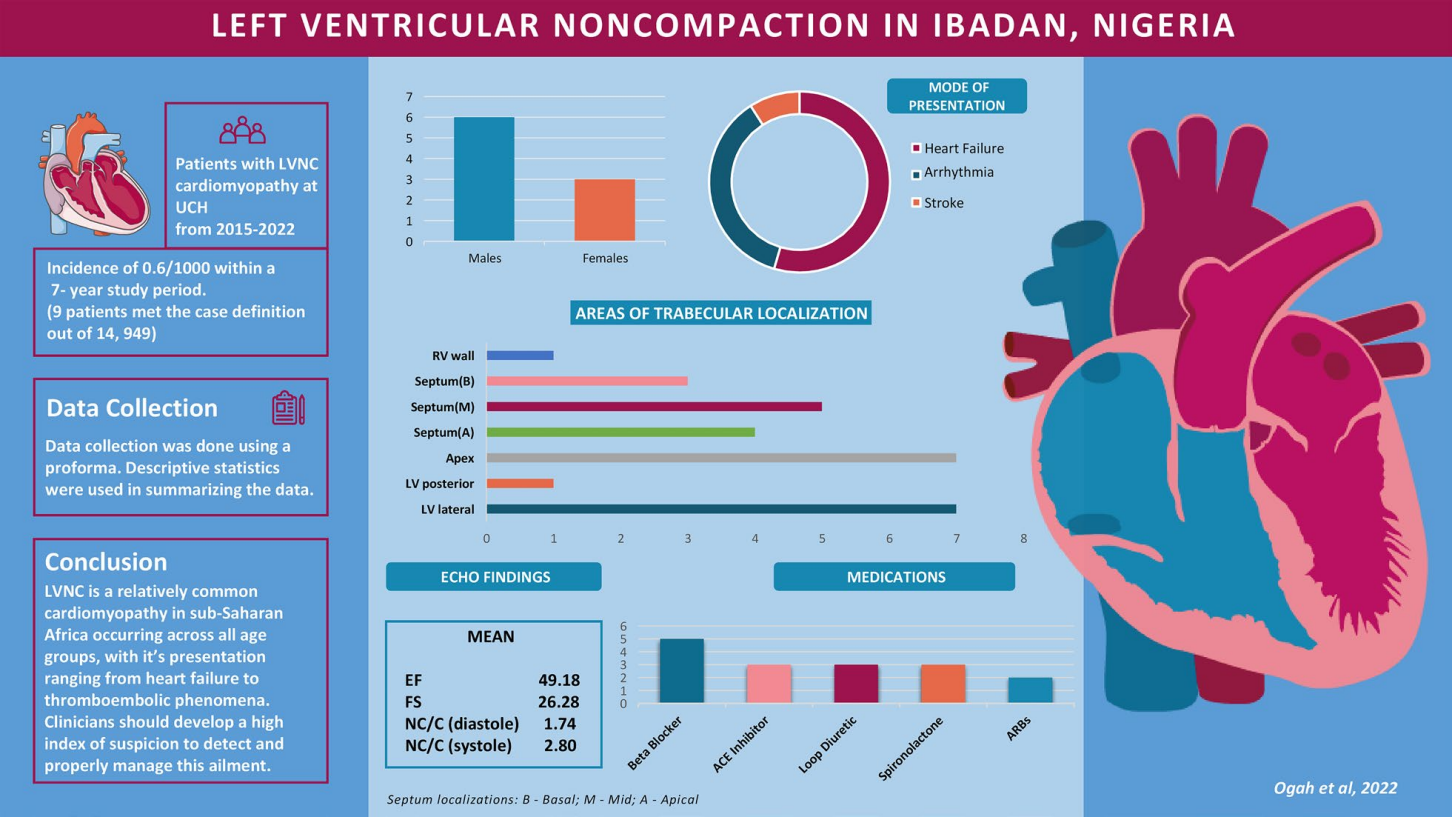
A graphic summary of the profile of the LVNC cases

LVNC is not uncommon in sub-Saharan Africa and presents in various ways, ranging from heart failure to arrhythmias and thromboembolic phenomena. It occurs across all age groups and is more common in males. Because the presentation is similar to other cardiac diseases, a high index of suspicion is required. The first-line modality for diagnosis is two-dimensional trans-thoracic echocardiography, where numerous trabeculations are seen, most commonly at the apex of the left ventricle, with a noncompacted/compacted ratio at end-systole greater than 2.0. Other findings which may be seen on echocardiography in patients with LVNC include valvular defects and wall motion abnormalities. Treatment varies, depending on the mode of presentation and the existing comorbidities (Fig. 3).

## Data Availability

The analysed datasets are available from the corresponding author on request.

## Abbreviations

ACE: Angiotensin converting enzyme
CMRI: Cardiac magnetic resonance imaging
ECG: Electrocardiogram
LV: Left ventricle
LVNC: Left ventricular noncompaction cardiomyopathy
NC/C: Noncompaction/compaction ratio
RV: Right ventricle
TAPSE: Tricuspid annular plane systolic excursion
ZASP: Z-band alternatively spliced PDZ-motif protein
